# A hierarchical architecture for recognising intentionality in mental tasks on a brain-computer interface

**DOI:** 10.1371/journal.pone.0218181

**Published:** 2019-06-18

**Authors:** Asier Salazar-Ramirez, Jose I. Martin, Raquel Martinez, Andoni Arruti, Javier Muguerza, Basilio Sierra

**Affiliations:** 1 Department of Computer Architecture and Technology, University of the Basque Country (UPV/EHU), Donostia-San Sebastián, Spain; 2 Department of System Engineering and Automation, University of the Basque Country (UPV/EHU), Bilbao, Spain; 3 Department of Computer Science and Artificial Intelligence, University of the Basque Country (UPV/EHU), Donostia-San Sebastián, Spain; Politechnika Krakowska im Tadeusza Kosciuszki, POLAND

## Abstract

A brain-computer interface (BCI), based on motor imagery EEG, uses information extracted from the electroencephalography signals generated by a person who intends to perform any action. One of the most important issues of current research is how to detect automatically whether the user intends to send some message to a certain device. This study presents a proposal, based on a hierarchical structured system, for recognising intentional and non-intentional mental tasks on a BCI system by applying machine learning techniques to the EEG signals. First-level clustering is performed to distinguish between intentional control (IC) and non-intentional control (NC) state patterns. Then, the patterns recognised as IC are passed on to a second stage where supervised learning techniques are used to classify them. In BCI applications, it is critical to correctly classify NC states with a low false positive rate (FPR) to avoid undesirable effects. According to the literature, we selected a maximum FPR of 10%. Under these conditions, our proposal achieved an average test accuracy of 66.6%, with an 8.2% FPR, for the BCI competition IIIa dataset. The main contribution of this paper is the hierarchical approach, based on machine learning paradigms, which performs intentional and non-intentional discrimination and, depending on the case, classifies the intended command selected by the user.

## Introduction

It was in 1924 when Hans Berger achieved to record the first human electroencephalogram [1]. Since then, the study of the brain has been a matter of interest for scientists, researchers and medical professionals. Related to the study of the activity of the brain, in the 1970s, researchers of the field of engineering began to show interest on brain activity and started producing the first Brain-Computer Interface (BCI) applications. In the early days of BCI, these applications had mainly a medical background and were focused on restoring either lost audition/visibility capabilities or mobility by allowing users to interact with a computer using their thoughts. However, the span covered by BCI nowadays has vastly increased and it is not only focused on medical applications but also to other applications from different fields, such as assistive technologies for elder people, videogame and entertainment, smart home control or even in for military applications.

Nevertheless, the interaction with computers via thinking is far from being a trivial problem as it requires monitoring and processing of the user’s brain activity. To do so, the electrical activity of the brain has to be captured by means of sensors. There are different ways to capture the activity of the brain [2]: electroencephalography (EEG), magneto encephalography, functional magnetic resonance imaging and, most recently, near-infrared spectroscopy. Anyway, independently from the signal capturing methodology, the signals coming from the brain have to be passed on to the computer that will be in charge of processing the information and deciding whether any specific action has to be taken or not [3], [4]. To get the answer to this question, the computer will analyse the characteristics of those signals and compare them to some specific patterns related to the actions to be taken. Finally, the computer will send a command to a final actuator or software application if it the decides that the signals match one of those patterns.

When choosing the operation mode of BCI systems, an option is to work with synchronous protocols in which the user of the system changes between the desired mental control states following a specific repetitive pattern [5], [6]. Therefore, synchronous systems are based on the recognition of EEG events that are tied to specific cues. Nevertheless, it is important to consider that the brain is not continuously sending orders to the limbs: a person can maintain the limbs within a state of inaction or change voluntarily between control states without needing to go through any other state in between [7]. Bearing this in mind, any BCI should be able to differentiate between intentional control (IC) states (when the user produces a desired control order to activate certain activity on the actuators) and non-intentional control (NC) states (the time period during which no desired control orders are produced) so that false responses are avoided [8]. In addition, the system should be able to distinguish among the different EEG patterns for IC states to relate each of them to the activation of a specific activity. Among the research works found in the literature, the authors in [9] consider the design of a self-paced BCI as a pattern rejection problem, where NC states must be rejected by the BCI, whereas IC states must be accepted and properly classified. Similar strategies are used in the referenced works [10–12]. They define a threshold that the signal must reach to indicate activity detection.

Thus, as previously explained, the problem can be divided into two classification tasks: a primary binary classification between IC states and NC states (positive and negative classes, respectively) and a secondary classification in which the IC patterns are assigned to a specific limb movement class. Considering this, it is crucial to minimise the false positive rate (FPR) during the primary classification, as false positive detections (FP, when an NC state is classified as IC) would lead to undesired actuator activities ([9], [12], [13]). Moreover, undesired system actuation due to a high FPR would also produce user frustration, which would subsequently make the BCI system not usable (for instance, no one would use a wheelchair that moved differently than desired due to the risks this may produce). The FPR is defined as the ratio between FP and the sum of FP and true negatives (TNs, the NC states that have not been classified as IC): *FPR* = *FP*/(*FP* + *TN*). Thus, considering the importance of minimising the FPR, different research has established a limit of 10% as the maximum permissible FPR value at which to consider a BCI as being a feasible tool ([9], [14], [15]).

This work is focused on designing a hierarchical system that uses a combination of several machine learning algorithms to classify motor imagination of movements where a mental process carried out by an individual simulates a given action. The motor imagery task is one of the most studied types of BCI systems [16–19]. The main contribution presented in this article is to address the imaginary motor classification problem based on a two-level hierarchical structure that combines both supervised and unsupervised algorithms. The goal of the first-level classification is to distinguish whether the user EEG is producing an IC. After that, if the first level classification corresponds to an IC pattern, then that pattern enters the second level classification to determine to which mental task the pattern belongs, i.e., right hand, left hand, foot or tongue imaginary movement. For the first level classification, the authors propose to cluster the EEG patterns by using a K-means algorithm in combination with a thresholding function. For the second level classification, the proposal is to use a support vector machine classifier. This hierarchical proposal maintains the FPR below 10%. The data used, the approaches explored and the applied methodology presented in this work are an extension of a preliminary study included in [20].

First, in section “Materials and methods”, this article gives an explanation of the experimental protocol that was followed for acquiring the data and for pre-processing it. In addition, this section also explains the previous two approaches that used only supervised algorithms and, after that, presents the proposal of the authors that includes unsupervised classification to enhance the performance of the imaginary motor classification. The experimental results of the research are presented in section “Experimental results (test dataset)”, and in “Discussion” section, those results will be discussed in comparison to those obtained by other studies in the literature. Finally, the authors will present the conclusions of the research and references will be given.

## Materials and methods

### Data set and experimental methodology

#### New data: Modification of the original database

For this study, the authors used data from the BCI competition III (specifically, the IIIa dataset) [21]. This dataset has already been used several times for benchmark evaluations, and its ease of accessibility makes it well suited for the reliability and reproducibility of the presented work.

The data of this dataset came from 3 volunteers named K3b, K6b and L1b. 60 electrodes were placed on the scalp of the volunteers to capture the EEG data at a sampling rate of 250 Hz. The data was collected while the subjects were sitting and staring at a computer; they had to produce imaginary movements of a single body part (right hand, left hand, foot or tongue) as a response to a random cue shown on the computer screen. For every instance, the subjects started watching a blank computer screen. At t = 2 s, a cross (“+”) would appear on the screen and a beep would be played to capture the attention of the subject. Then, 1 s later (at t = 3 s), an arrow would appear in addition to the cross; the arrow would remain for 1 s pointing to the direction corresponding to the limb with which the participant had to perform an imaginary movement. The imaginary movement had to last for 3 seconds: from the appearance of the arrow until the “+” cross disappeared from the screen (from t = 4 s to t = 7 s). Then, participants were given a 2 s break before the next imaginary movement attempt. Once all the trials had been recorded, the EEG signals were band filtered to the 1-50Hz range and, later, notch filtered at 50Hz to remove any possible noise coming from the power grid.

**Fig 1:**
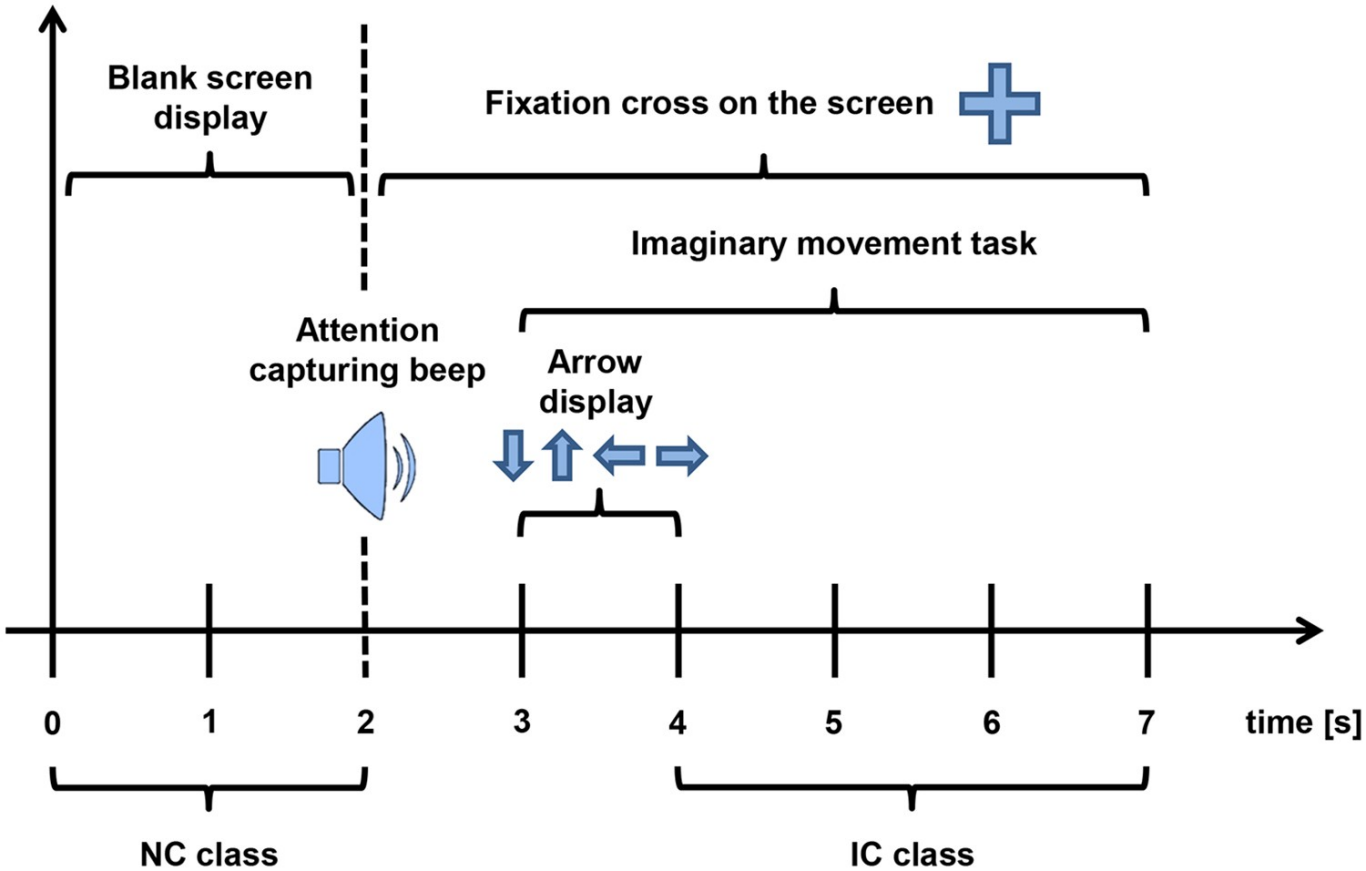
Structure of a imaginary movement trials.

After the collection process (the structure of one of these imaginary movement trials is depicted in Fig 1), the dataset contained 840 cases (360 for K3b and 240 for K6b and L1b), all of which were labelled for a single body part class. The number of cases for each of the four classes is equal, so the database has a balanced class distribution.

Although the imaginary movements were time locked to the cues, to make the problem closer to a real-life operation, the researchers decided to include those instances belonging to the intervals previous to the cues in the study. In this way, the classifiers not only have to distinguish among different IC patterns but also must be able to differentiate between intended and unintended control brain activities. When it comes to including new data to build the NC class it is important that the data is not biased by any outer stimuli. In this sense, it might be possible to think that the interval from 0-3 s would be good to feed this class, but both the fixation cross and the attention capturing beep could bias the data. Thus, similarly to what done in [22], only the data related to the 0-2 s time intervals have been labelled as belonging to a new NC class as they are clean from any outer stimuli. As a consequence of adding this new class, the database increased in 240 instances for participant K3b and in 160 instances for K6b and L1b. Therefore, the total number of instances ended being 600 for K3b (360 IC and 240 NC), 400 for K6b (240 IC and 160 NC) and L1b (240 IC and 160 NC).

#### Database pre-processing, feature extraction and dataset preparation

After modifying the database, the team considered the work presented in [23] and decided to apply the common spatial patterns method (CSP) [24], [25] to the original set of 60 EEG signals. The application of this technique gives as result a projection matrix that permits to obtain a set of transformed signals (also called projections) sorted according to their meaningfulness for predicting an instance as belonging to a target class. As there are 5 different classes, in this study the technique was applied 5 times following a pair-wise approach (once per class: 1 NC and 4 IC), subsequently obtaining 5 different projection matrices. Thus, for each class a new set of 60 rearranged projections was calculated. Later, only the first 5 projections of each class were selected (the most representative) and then they were band filtered into three different frequency bands: 8-12 Hz, 12-20 Hz and 20-30 Hz.

After pre-processing, the data went through the feature extraction process. We considered 7 features to be of interest (see Table 1): minimum and maximum voltage values, mean voltage, voltage range, average power of the signals, rate of zero voltage crossings and rate of samples above zero volts. As a result of the whole pre-processing, a total of 525 numerical values were obtained for each instance in the database (5 classes x 5 projections x 3 filter bands x 7 meaningful features).

**Table 1:** Mathematical descriptions of the extracted 7 features.

Extracted features	
Minimum voltage	*V*_min_ = *min*(*X*_n_)
Maximum voltage	*V*_max_ = *max*(*X*_*n*_)
Mean voltage	$V_{\text{mean}}=\frac{1}{N}\cdot \sum_{n=1}^{N}X_{n}$
Voltage range	*V*_mean_ = *V*_max_ − *V*_min_
Average signal power	$P_{\text{signal}}=\frac{1}{N}\cdot \sum_{n=1}^{N}X_{n}^{2}$
Rate 0 voltage crossings	$R_{0-\text{cross}}=\frac{1}{N-1}\cdot \sum_{n=1}^{N}\left[sign\left(X_{n}-1\right)\neq sign\left(X_{n}\right)\right]$
Rate of samples above 0 volts	$R_{\text{positive}}=\frac{1}{N}\cdot \sum_{n=1}^{N}\left(X_{n}\geq 0\right)$

Once the data was conditioned, it was randomly divided for each of the subjects into three smaller datasets: TrainingSet1, TrainingSet2 and TestSet. These three datasets would be used for the training and testing (as their names state) of the proposed two-level hierarchical system. For subject K3b, the datasets were as follows: TrainingSet1, contained 240 instances (30 for each of the IC classes and 120 corresponding to NC); TrainingSet2, had 120 instances (all of them IC, distributed equally as 30 instances per class); and finally, TestSet, had 120 cases for NC and 30 instances for each of the IC classes. The datasets of the other two subjects contained 160, 80 and 160 instances, respectively (the proportions between all the classes in each set was the same as for K3b).

This process of randomly separating the data into three independent datasets was repeated 5 times. It has been done this way in order to attempt 5 runs for training and testing the different approaches presented later the following sections of the article. On the one hand, all the TrainingSet1 and TrainingSet2 have been used to respectively train the first and second levels of the hierarchical system following the 10-fold cross-validation methodology. Consequently, the values that will be given in the tables showing the results for the training phases will correspond to the average values of the 50 estimations obtained from this 5-run 10-fold cross-validation strategy. On the other hand, all TestSet data have been used to test the final performance of the system and the results presented in tables for the test phase are representative for the average scores obtained from the test 5 runs. Moreover, with the intention of giving more consistent information, the result tables of the test phase will also give the standard deviations obtained from those 5 runs.

Finally, each instance having so many features could be troublesome due to the curse of dimensionality (a frequent problem in classification tasks that typically have data from a small number of training instances but whose dimensions are too large to handle effectively). Therefore, it was necessary to reduce the dimensionality of the data to make the learning algorithm simpler. Looking to dismiss the features that provide useless information or that are redundant, the researchers decided to apply the correlation-based feature selection method [26], as was also done in [23]. What this method does is to look for the features that correlate best to a single class while at the same time they are very weakly correlated to the other classes. We applied this technique by means of the Weka platform [27]: we started with a blank feature set and added new features using a greedy search option (best first). Only the training data was used to determine which features to discard for being less useful. However, the same features discarded from the training data would be removed from the testing datasets in order to make the dimensions of the instances entering the classifier coherent in both training and testing processes. After this process, the dataset instances ended having an average amount of 65, 31 and 36 features over the 5 runs for subjects K3B, L1b and K6b, respectively.

**Fig 2:**
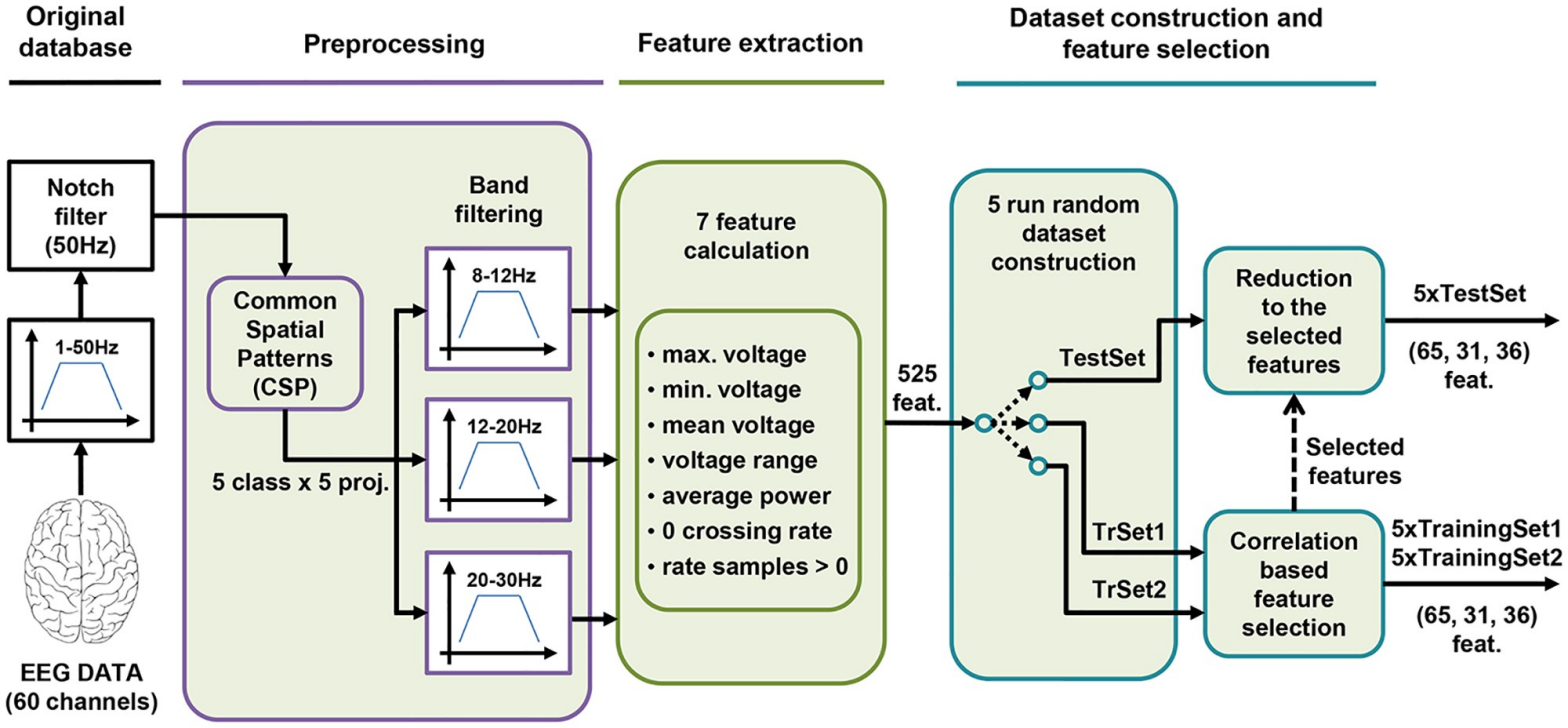
Diagram of the whole construction process of the new datasets.

So, as a summary, first the EEG signals were rearranged using the CSP method and only the first 5 projections for each class were passed onto the pre-processing stage in which these projections were separated in three different frequency bands. Later, the previously mentioned 7 features were extracted for each of those bands (getting S1 Dataset which is included in the “Supporting information” section). These went through 5-runs of random data partition and dataset building process in order to build 5 different datasets for training (TrainingSet1 and TrainingSet2) and testing (TestSet) the hierarchical classifying system presented in this work. Finally, the correlation based feature selection method was used to reduce the dimensionality the dataset instances, getting as a result the new datasets used to train and test the proposed hierarchical classifier (TrainingSet1, TrainingSet2 and TestSet). Fig 2 presents a diagram summarizing the whole pre-processing and data preparation process.

### Previous approaches

This subsection presents two approaches using only supervised learning algorithms: (a) a one-level system composed of a classifier dealing with the five classes (NC, right hand, left hand, tongue and foot imaginary movement) and (b) a two-level system, introducing first a classifier with the aim of distinguishing between IC and NC signals and presenting only the cases labelled as IC in the first level of the system to the four-class classifier of the second level.

The goal of this analysis is to show the limitations that the approaches solely based on supervised learning methods have in keeping the FPR below 10%. Therefore, in this work we will present a new system based on unsupervised learning algorithms that is able to maintain the FPR below this threshold.

#### One-level system

As previously mentioned, the first approach was to classify all states within a single stage and was conducted using supervised learning algorithms. To date, several algorithms have been used in the literature related to BCI systems [28], [29]. Among them, we opted for the following 11 algorithms: decision trees (DTs), random forests (RFs), a 10 decision tree combination of Ada boost (AdaB) and bagging (Bag) algorithms, logistic regression (LR), k-nearest neighbours (specifically 1-NN and 5-NN), support vector machines (SVMs), the 1R rule, a radial-basis function (RBF) network and naïve Bayes (NB). We have chosen these algorithms because this selection is representative of the machine learning state of the art and because it includes approaches from different machine learning paradigms (algorithms based on rules, on trees, on distances, probabilistic methods, function-based algorithms and ensembles of classifiers). Initially, the team tried to fine-tune the settings of the algorithms. Anyway, after seeing that changing the settings was most likely to improve the performance for some of the subjects at the expense of a loss for the others, the final implementation of all the listed algorithms was performed using the structures and parameters that come by default in Weka software.

We analysed the performance of these classification algorithms to develop the one-level classifier for the five classes (NC, left hand, right hand, tongue or foot). All of the classifiers were trained using part of the TrainingSet1 and TrainingSet2 datasets: 300 cases (60 cases of each of the five classes) for subject K3b and 200 cases (40 cases of each class) for subjects L1b and K6b. Table 2 shows the accuracy of each classifier for the three subjects. On average, the best overall classifier was the SVM algorithm, with an accuracy of 72.3%.

**Table 2:** Performance classification of a one-level system.

		Estimation of the best classifier											Test
		1R	DT	1-NN	5-NN	NB	RBF	SVM	LR	AdaB	Bag	RF	SVM
K3b	Accuracy (%)	39.1	64.8	72.2	75.6	75.7	77.0	81.6*	67.7	75.8	71.9	73.0	77.5±3.7
	FPR (%)	75.0	52.0	48.3	48.7	35.7	33.0	31.6	42.3	37.3	44.0	47.0	29.2±4.9
L1b	Accuracy (%)	45.8	62.6	62.8	70.3	70.3	70.5	73.5*	60.3	69.3	68.7	68.4	74.4±3.2
	FPR (%)	79.0	50.5	48.5	41.5	45.0	37.0	34.0	42.5	42.0	41.5	45.5	27.5±7.2
K6b	Accuracy (%)	35.5	47.7	49.9	52.8	51.3	57.1	57.3*	48.5	56.2	56.1	55.4	55.6±3.9
	FPR (%)	55.0	55.0	51.0	55.0	45.0	41.0	44.5	53.0	41.5	38.5	39.0	36.2±10.0
Aver.	Accuracy (%)	40.0	59.3	63.1	67.6	67.2	69.5	72.3*	60.1	68.3	66.5	66.6	70.4±3.6
	FPR (%)	70.4	52.4	49.1	48.4	41.0	36.4	36.0	45.4	39.9	41.7	44.3	30.7±7.0

* shows the highest accuracy for each subject when estimating the best classifier using 5-run, 10-CV. The standard deviation is also given for the test results.

We applied the nonparametric Wilcoxon test [30], with a 95% significance level, to determine if there are statistically significant differences between the accuracy of the analysed algorithms. The test shows that the differences between the accuracy of the SVM classifier and the rest of the classifiers are statistically significant, in favour of the SVM classifier (with p-values of 0.02).

Therefore, the performance of the SVM-based system was tested with the TestSet datasets (last column of Table 2). The accuracy obtained using the SVM classifier was 70.4% on average for the three subjects. The main problem of this approach is that the FPR (average 30.7%) is above the desirable threshold (10%). This makes the system unfeasible from a practical point of view.

#### Two-level system based on supervised learning

With the aim of improving the accuracy, or at least maintaining it, but keeping the FPR below 10%, we built a two-level hierarchical system based only on supervised learning. The first level is specialised to distinguish between NC and IC states without distinguishing between the four possible IC states. The second level will classify between the four possible IC states only the cases labelled as IC by the first level of the system.

We used the same classifiers we selected to build the one-level system. Table 3 shows the results obtained using TrainingSet1 in the estimations of the first level for each classifier and subject considered. In all cases, the FPR is above 10%. The best averaged result is obtained by the 5-NN classifier with an average accuracy for the three subjects of 82.4% and an FPR of 16.4%. Table 4 shows the accuracy achieved by each classifier for the three subjects and the average results in the estimation of the second level classifier using TrainingSet2 datasets. The best overall classifier was the SVM algorithm, with an average accuracy of 78.2%.

**Table 3:** Estimation of the best classifier for the first level in the two-level system with supervised classifiers: Accuracy and FPR.

		1R	DT	1-NN	5-NN	NB	RBF	SVM	LR	AdaB	Bag	RF
K3b	Accuracy (%)	66.9	71.9	79.6	84.0*	78.3	80.2	81.8	73.7	79.7	78.7	78.4
	FPR (%)	34.0	28.5	22.5	12.8	26.2	19.5	17.5	25.7	21.3	20.3	24.8
L1b	Accuracy (%)	71.4	75.5	79.9	82.5	77.1	79.9	81.1	80.5	81.3	80.4	83.1*
	FPR (%)	30.8	24.3	18.8	17.0	38.8	31.5	25.5	18.8	19.5	22.3	22.8
K6b	Accuracy (%)	76.6	75.5	76.9	80.0	79.4	81.5	82.1	76.5	81.0	82.4*	81.8
	FPR (%)	20.3	24.8	25.5	21.0	22.5	15.3	18.3	24.5	18.0	16.8	20.3
Aver.	Accuracy (%)	71.0	74.0	78.9	82.4*	78.3	80.5	81.7	76.4	80.5	80.2	80.7
	FPR (%)	29.1	26.2	22.3	16.4	28.7	21.7	20.0	23.4	19.9	19.9	22.9

The values are the average for the 5-runs using 10-fold CV, representing * the highest accuracy for each subject.

**Table 4:** Estimation of the best classifier for the second level in the two-level system with supervised classifiers: Accuracy.

Accuracy (%)	1R	DT	1-NN	5-NN	NB	RBF	SVM	LR	AdaB	Bag	RF
K3b	46.5	69.0	79.2	83.5	81.2	81.2	88.3*	69.7	79.2	75.8	81.5
L1b	54.8	68.5	66.3	70.3	74.5	71.3	80.8*	69.8	75.5	73.3	73.5
K6b	35.8	57.5	51.0	57.5	54.5	55.5	60.3*	49.8	59.0	57.8	56.5
Average	45.8	65.6	67.4	72.3	71.7	71.0	78.2*	64.0	72.4	69.9	72.1

The values are the average for the 5-runs using 10-fold CV, representing * the highest accuracy for each subject.

As in the previous subsection, we applied the Wilcoxon test (95%) to analyse the existence of statistically significant differences in the accuracy of the classifiers. In the first level of the system, the test shows that there are no statistically significant differences between the 5-NN classifier and the others. However, at the second level, there are statistically significant differences in favour of the SVM classifier (with p-value of 0.02).

The results of this system for the test sets (TestSet datasets) corroborate the results obtained with the training datasets. The average FPR is 18.2(±5.6)%, which is still above the maximum threshold allowed for this type of application, and the average accuracy for the whole system was 71.2(±3.3)%.

In summary, we were not able to find a system based only on supervised learning algorithms to differentiate between the four IC states that maintained the FPR under 10% using Weka’s default settings. However, the team explored fine-tuned options for the systems that obtained the best performances in both topologies: a fine-tuned single level SVM classifying system and a two level classifying system using 5-NN and SVM supervised learning algorithms. In both cases the team tuned the threshold used to determine whether an instance belongs to the IC classes, getting as result systems with FPR ratios below the required 10%. Nevertheless, in both cases the overall accuracy of the system decreased drastically, being lower than the accuracy of hierarchical model proposed in this work. As a consequence, we decided to apply an unsupervised learning algorithm at the first level for the system looking to group NC and IC states in different clusters. This way, it is possible to define a minimum proportion of IC cases (IC-threshold) in a cluster to label this cluster as IC class. The next subsection presents this new proposal.

### Unsupervised learning-based hierarchical classifier

**Fig 3:**
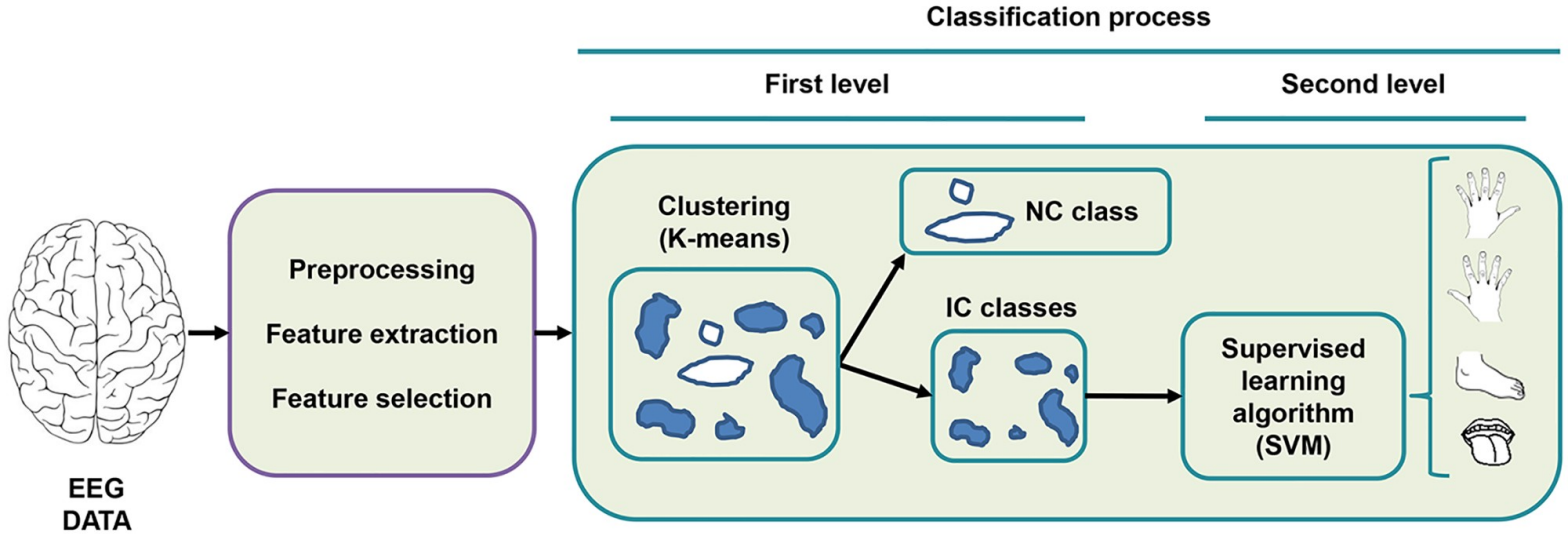
Structure of the two-level hierarchical classification system.

The new proposal also has a hierarchical structure. The first level determines the presence or absence of intentional activity in the EEG signal by applying clustering techniques and a threshold function. The second level determines whether the detected intentional activity is a left hand, right hand, tongue or foot imaginary movement. Fig 3 shows a diagram that present how this two level hierarchical classification process is done.

#### First level: Intentionality detection

For determining whether an instance belonged to the IC or NC class, i.e., for the first level classification, we used the Euclidean distance K-means algorithm included in Weka. Once the instances had been clustered, each of the clusters went through a labelling process in which they were labelled as NC or IC setting as a constraint that the FPR had to always be below 10%. To help fulfil this condition, we applied a strategy similar to that proposed in [31]: every cluster labelled as IC had to contain at least a minimum percentage of IC instances (named the IC threshold).

Having defined the strategy to follow, it was necessary to determine the K value of the K-means algorithm and the IC threshold for the IC clusters. The training was performed using 12 values for K: 5, 10, 15, 20, 25, 30, 35, 40, 45, 50, 55 and 60. The training was performed using dataset TrainingSet1 and applying the 5-run 10-fold cross validation methodology and setting the average linkage distance as the criterion for assigning the test instances to the previously defined clusters. The training was repeated using different IC-threshold percentages for labelling a cluster as belonging to an IC class.

**Fig 4:**
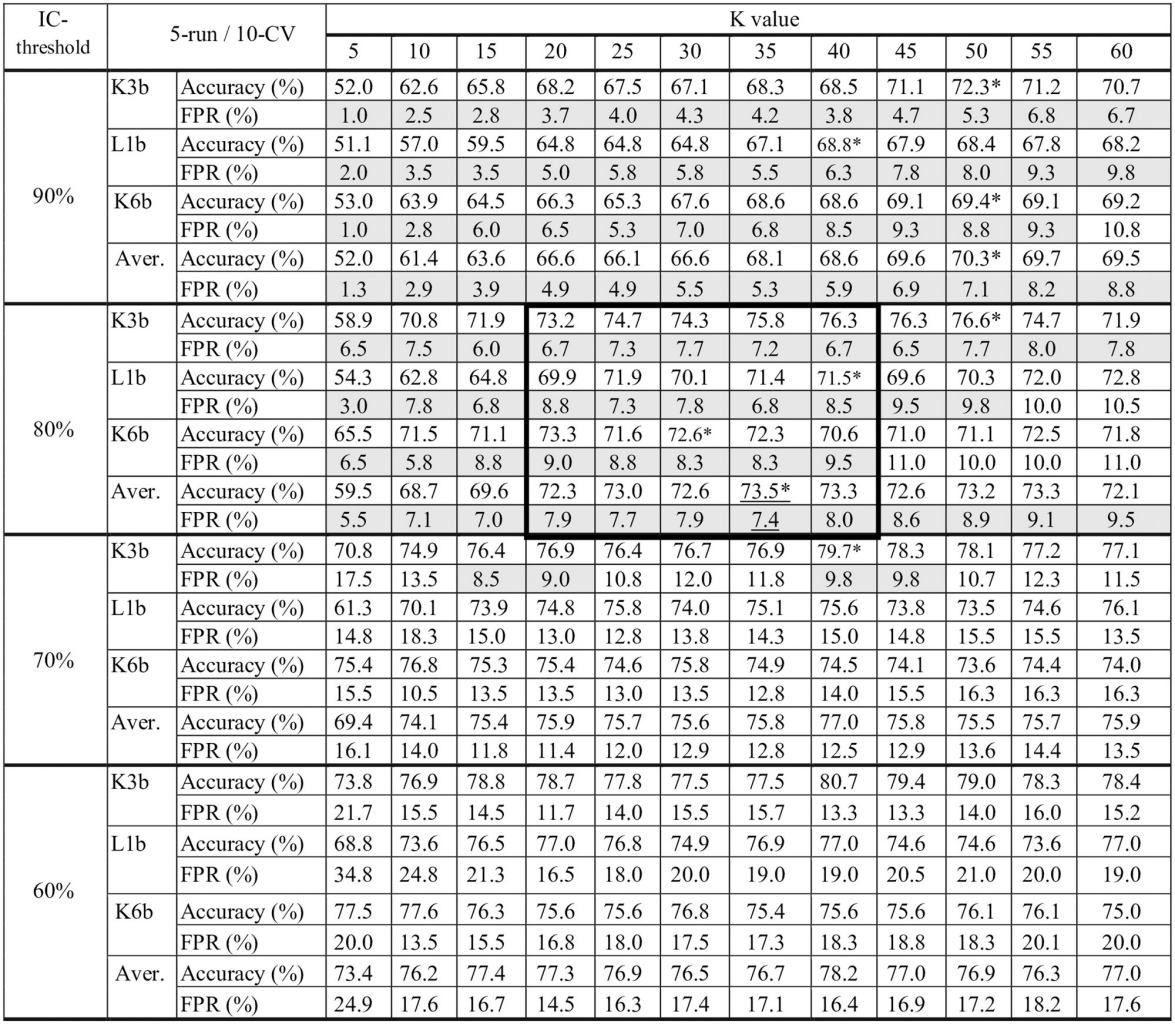
Estimation of the K value and IC threshold: Accuracy and FPR depending on the value of the K parameter and the IC threshold. The values are the average for the 5-runs using 10-fold CV. Those values with FPR below 10% are shaded in grey, * indicates the highest accuracies, and the best options are underlined bold letters. The values within the bold box show cases without statistically significant differences in accuracy.

Fig 4 shows the average results obtained per subject, and the results averaged for the three subjects. Taking into account these average results, the best performance is obtained for K = 35 and an IC-threshold of 80%, with an accuracy of 73.5% and an FPR of 7.4%. With a high IC threshold, the accuracy decreases, whereas with a low IC threshold, the FPR increases. Thus, the IC threshold was selected from a range of appropriate values for the FPR rate, maintaining this ratio below 10% and maximising the accuracy. The best option is one in which the highest average accuracy for the three subjects is obtained while maintaining the FPR below 10%.

After applying the analysis of statistically significant differences in relation to the obtained accuracy, a value of 80% for the IC threshold shows statistically significant differences with the rest of the tested values. Regarding the K parameter, we found several values without statistically significant differences, ranging from 20 to 40 (K = 35 is the option that maximises and minimises respectively the average values of accuracy and FPR); however, this range is statistically significantly different from the rest of the K values. We applied the Wilcoxon test with a 95% significance level. The p-values obtained are lower than 0.02 in all cases.

#### Second level: Intentional action classification

After seeing that setting an IC-threshold of 80% and a K = 35 obtained the best results for the first level classification, the team had to choose the best algorithm for the second level classification. For the second level classification, we considered only supervised learning algorithms. According to the results obtained in the previous experiments presented (see Table 4), the best overall classifier was the SVM algorithm, with an average accuracy of 78.2%. Therefore, the team opted for this algorithm.

To summarise, the hierarchical BCI system proposed in this work consists of a combination of a first level classifier based on a distance thresholding K-means clustering and an SVM as the second level classifier. Whereas the first level classifier is used to distinguish between IC and NC classes, the second classifier is used only to determine the different classes within the clusters labelled IC. This approach maintains the FPR under the reference threshold of 10%, as explained in the following section, where the results obtained for the test dataset (TestSet) will be presented.

## Experimental results (test dataset)

As previously mentioned, the final step of the design process is to verify that the system is able to differentiate between classes for new EEG signal patterns. To do so, new data that the system has not previously used is needed, i.e., the test datasets (TestSet) that had previously been obtained from the 5 repetitions of the random dataset construction process. Therefore, the average results of applying the first level classification to TestSet over 5 runs are presented in Table 5, where the following information is given: the average confusion matrix, the FPR and the accuracy of the primary classification.

**Table 5:** Classification performance for the first level using the TestSet dataset.

(K-means, K = 35) (IC-threshold 80%)	Confusion Matrix			FPR (%)	Accuracy (%)
K3b		IC-estimated	NC-estimated	7.2±4.1	73.5±2.9
	IC-real	65	55		
	NC-real	9	11		
L1b		IC-estimated	NC-estimated	8.5±3.6	74.3±5.8
	IC-real	46	34		
	NC-real	7	73		
K6b		IC-estimated	NC-estimated	9.5±4.2	70.5±2.9
	IC-real	40	40		
	NC-real	8	72		
**Average**				**8.2**±3.9	**72.9**±3.7

The values are the average results for the test over 5-runs. The standard deviations are also given.

First, for subject K3b, Table 5 shows that the system failed to classify 9 NC instances that were considered to correspond to one of the four IC movements. This misclassification produced a 7.2% FPR. In addition, from the total 120 instances, 65 of them would be passed to the second level classifier as they had been considered to belong to an IC class. With regard to subject L1b and K6b, 7 and 8 NC instances were incorrectly classified, leading to 8.5% and 9.5% FPR values, respectively. Subjects L1b and K6b had 120 initial instances, of which 46 and 40 were classified as IC patterns, respectively. These instances were subsequently selected to go through the second level classification. The FPR average rate of the classifier is 8.2%, within the limits of the application.

After the first classification, the patterns clustered as IC were passed to the second level SVM classifier. During this second classification, these instances were classified for one of the four possible imaginary movements: right hand, left hand, foot and tongue. Table 6 presents both the average confusion matrix and accuracies for the three subjects (89.2%, 78.3% and 57.5%, respectively). Regarding the confusion matrix, it can be seen that the biggest problem for the BCI system was to distinguish between the two hands. In the case of subject K6b, tongue movement achieved the poorest accuracy.

**Table 6:** Classification performance for the second level with the TestSet dataset.

	Confusion Matrix					Accuracy (%)
K3b		Left-estimated	Right-estimated	Tongue-estimated	Foot-estimated	89.2±2.0
	Left-real	11	3	0	0	
	Right-real	2	21	0	0	
	Tongue-real	0	0	12	1	
	Foot-real	0	0	1	14	
L1b		Left-estimated	Right-estimated	Tongue-estimated	Foot-estimated	78.3±1.4
	Left-real	9	1	0	0	
	Right-real	2	12	0	0	
	Tongue-real	1	1	5	3	
	Foot-real	0	0	2	10	
K6b		Left-estimated	Right-estimated	Tongue-estimated	Foot-estimated	57.5±8.5
	Left-real	6	5	0	0	
	Right-real	4	7	0	0	
	Tongue-real	2	3	2	0	
	Foot-real	1	1	1	8	

The values are the average results for the test over 5-runs. The standard deviation of the accuracy is also given.

Analysing the overall performance of the system in terms of classifying the five different patterns (NC class and 4 IC imaginary movements), the accuracy for subject K3b is 70.4%, 68.1% for subject L1b, and 59.4% for subject K6b (Table 7). These variations on the performance of the participants, being K3b the best and K6b the worst also had happened in [32]. This difference is produced because the three participants had been given different degrees of BCI training. Therefore, as stated in [23], the results obtained when classifying imaginary movement patterns vary considerably depending on the skillfulness of the participant. Whereas K6b was a total beginner, L1b and K3b had already had some experience with BCI (being K3b the one who had trained the most). Taking into account all subjects, the average accuracy of the system is 66.6%, with an FPR of 8.2%.

**Table 7:** Classification performance for the proposed system with the TestSet dataset.

	K3b	L1b	K6b	Average
FPR (%)	7.2±4.1	8.5±3.6	9.5±4.2	**8.2±3.9**
Accuracy (%)	70.4±2.2	68.1±2.0	59.4±3.7	**66.6±2.6**

The values are the average results for the test over 5-runs. The standard deviations are also given.

It is also important to analyse the times used by the system: if the time between epochs is shorter that the time needed by the system to classify then the use of the system would not be feasible. Thus, the team analysed the times needed both to train and to test the proposed two level hierarchical system. These times can be seen in Table 8. For the training, the times shown in Table 8 correspond, on the one hand, to the average time for a single training instance, and, on the other hand, to the average time needed to train the whole system. It is interesting to bear in mind that the clustering model and the SVM model can be generated independently and so, that it is possible to do create the models in parallel. With regard to the testing phase, only the average time per instance has been given. In addition, it is worth remembering that the feature selection time for the test datasets is negligible compared to the times shown in this table as the features would have already been selected during the training phase.

**Table 8:** Average training and testing times needed by the system.

Average (K3b / L1b / K6b)	Training	Training	Test
	Model	Instance	Instance
CSP	65.006	0.108	< 0.001
Feature extraction	5.246	0.009	0.009
Feature selection	13.337	0.022	-
K-mean	1.047	< 0.001	< 0.001
SVM	0.929	< 0.001	< 0.001
**Total time (s)**	**84.636**	**< 0.140**	**< 0.012**

The values given in Table 8 correspond to the average times needed for all the three subjects, using all the available data per subject to train the system by means of a machine using Windows 7 (64 bit) with an Intel Core i7-3770, 3.4 GHz processor. As it can be seen, the system needs 140 ms of training time per instance and it is possible to train the whole system in less than 2 min. Regarding the test, the system is able to classify a new pattern in 12 ms, which is a much shorter time compared to the window shifting time of the systems based on sliding window methods as the ones proposed in [12], [13] and [14], which use times between 0.5 s and 2 s and that will be further discussed in the next section.

Finally, we tried another variation of the system substituting the 4-class classifier of the second level with a 5-class classifier, including the NC class in the second level, to address the misclassified NC patterns in the first level of the system (as they have been misclassified as IC, they pass to the next level). This option allows us to be more relaxed with the IC threshold in the cluster labelling phase with more IC patterns arriving at the second level because the system was able to recover the misclassified NC patterns at the second level.

Table 9 shows these results for the test dataset, selecting some sub-optimal values of K and IC-threshold parameters for the first level. The global average accuracy increased by 3 points (from 66.6% to 69.8%), and the average FPR increased from 8.2% to 10.9%, especially for subject K6b (from 9.5% to 15%). Depending on the context of the application and the profile of the users (the degree of skilfulness), this option could be suitable to increase accuracy while maintaining the FPR under the desirable value for specific skilful users. For instance, for users with a profile similar to the K3b user, experienced users, the system could improve the accuracy 4 points while maintaining the FPR under 10%.

**Table 9:** Classification performance for the system with a 5-class classifier in the second level.

		K3b	L1b	K6b	Average
K = 20 IC-threshold = 70%	FPR (%)	8.5±2.6	8.5±4.2	14.5±7.4	**10.2±4.4**
	Accuracy (%)	73.9±3.4	69.1±3.6	61.1±5.3	**68.9±4.0**
K = 25 IC-threshold = 70%	FPR (%)	9.5±4.5	8.0±3.5	13.5±4.5	**10.2±4.2**
	Accuracy (%)	74.5±4.0	70.3±3.4	60.1±3.4	**69.2±3.7**
K = 35 IC-threshold = 70%	FPR (%)	10.5±5.9	7.5±3.9	15.0±5.4	**10.9±5.2**
	Accuracy (%)	74.9±2.6	70.6±3.6	61.1±3.7	**69.8±3.2**

The values are the average results for the test over 5-runs using TesSet dataset. The standard deviations are also given.

## Discussion

Despite the experimental setup of this work being slightly different from that of other research, it can be considered that the results obtained are similar to those presented in other studies in the literature. Table 10 summarises the information related to similar works found in the bibliography in the context of BCI systems. For each system, Table 10 shows the number of classes or motor imagery movements (and if the system considers the NC state, NC class), the false positive rate (FPR), the accuracy of the system and the algorithms used for building the system.

**Table 10:** Comparison to other related approaches.

Reference	Number of classes (+ NC class)	FPR (%)	Accuracy (%)	Algorithm employed
[23]	4 (+0)	—	74.2	Support Vector Machine
[33]	4 (+0)	—	64.4	Hierarchical Support Vector Machine
[34]	4 (+0)	—	77.6	Multi-class CSP + Fuzzy System
[9]	2 (+1)	10	83.4	Support Vector Machine
[12]	2 (+1)	26.7 / 28.3	73 / 75	ROC Curve Analysis
[13]	1 (+1)	17	72	Support Vector Machine
[14]	3 (+1)	19	84.3	Mahalanobis Linear Distance Classifier
[35]	2 (+1)	21.7	73.5	Correlation-Based
[36]	2 (+1)	1	54	k-Nearest Neighbour + Linear Discriminant Analysis
**Our approach**	4 (+1)	8.2	66.6	K-means + Support Vector Machine

On the one hand, there are works that do not consider the difficulty of including the NC state in the classification process. For instance, the work presented by [33] uses an approach based on a hierarchical support vector machine classifier and the BCI competition IV-IIa dataset with four imagery motor tasks. The system achieves an accuracy of 64.4%. The work in [34] achieves an accuracy of 77.6% for the data set IIIa (BCI competition III) using a fuzzy system combined with a multi-class extension of the CSP algorithm. Finally, [23] uses the same dataset but with only four classes (the NC class is not included in the work). They achieved an accuracy of 74.2% by applying an SVM classifier.

On the other hand, regarding the works that include the NC state, the work of [12] presents the results of experiments carried out with three subjects in a two-class problem (right/left motor imagery movements). Their system’s accuracy is approximately 75% for right movement and 73% for left movement, but in both cases, the FPR is greater than 10% (26.7% and 28.3%, respectively). The authors in [35] present results on a hand or foot movement intention detection problem. They achieved an accuracy of 73.5%, but with an FPR of 21.7%, using correlation-based classifiers. The work referenced in [13] presents an evaluation of seven classifiers in a problem of arm movement intention detection (one class problem). They consider 6 subjects in the experiments. The best results were obtained using an SVM classifier, with approximately 72% accuracy. As they stated, the problem is the FPR, which is above 17% (in some cases, it reached up to 75%). In these cases, when the user wants to remain at rest, if the classifier detects a movement, the user will perform an unintentional movement, which would be an inconvenience to the user. The same problem is noted in [14], where in the experimentation of 5 subjects, the achieved accuracy is 84.3%, but with an FPR of 19%.

In all the works mentioned above the FPR is over 10%, which is the maximum threshold allowed for this type of application. In contrast, the referenced work [9] obtained an accuracy of 83.4% for a two-class problem (only left or right hand movements) with an FPR of 10% using an SVM. Finally, the work referenced in [36] achieves an accuracy of 54%, with an FPR rate of 1% using a combination of k-NN and LDA classifiers. In both examples, the complexity of the particular problem is smaller than in our case because they deal with only two classes.

The results obtained show the appropriateness of the new paradigm, which could be used to develop efficient systems in several BCI applications. This research has extremely high impact, especially for motor disabled individuals (e.g., quadriplegic people), providing a non-muscular communication channel that allows them to interact in real world situations, such as guiding a wheelchair, moving a pointer, prosthetic limb control, and controlling any kind of device [14], [19], [37].

## Conclusions

This paper presents a BCI system that is capable of distinguishing between intentional and non-intentional control (IC and NC) mental states. In addition, the system is capable of determining the specific imaginary movement of those mental states considered to be IC. The system divides the problem hierarchically: first, the mental patterns are classified with an unsupervised clustering algorithm that determines whether the pattern belongs to an IC or an NC state. Second, a supervised learning algorithm decides the specific imaginary movement class of the patterns that were classified as IC states (left hand, right hand, tongue and foot).

The dataset used during the design process was the dataset IIIa from the well-known BCI competition III. The database was slightly modified by including in the data the first three seconds of each of the attempts as belonging to an NC state. Then, the database was pre-processed to extract and select the most meaningful features. Later, the data went through the previously mentioned two level classifiers: a K-means clustering algorithm performed the primary classification, and the differentiation between IC classes was determined by applying a support vector machine (SVM). The experiment was carried out using the 5-run, 10-fold cross-validation methodology.

The performance achieved by the system was different for the analysed subjects; therefore, the skill of the users with this kind of system seems to influence the final results. On average, for all subjects, the overall accuracy is 66.6%, using an independent test data set. These results were obtained while maintaining the false positive rate (FPR) under 10% (achieving an average 8.2% rate for the subjects participating in the experiment). The robust detection of motor intention is a crucial issue for the development of BCI control systems.

We have compared the final approach with other alternatives that use only supervised algorithms, with and without a hierarchical structure, and using 4- or 5-class classifiers. In all these variants, the FPR obtained is above the maximum limit. This fact confirms the advantage of using a hierarchical approach based on unsupervised learning in comparison with other analysed approaches. It is also able to maintain the FPR below 10%, a critical condition in these applications. A high FPR tends to cause undesirable effects, making the resulting BCI system unusable in real world situations (e.g., undesirable wheelchair movements that could be dangerous for the user).

As future work, we plan to apply this approach to non-segmented data, that is, to build a self-paced BCI system [38], [39] using a sliding time window technique and analysing the influence of the overlap among the windows in the performance of the system. Moreover, we intend to use automatic channel selection techniques [40], [41] to reduce the dimensionality and to build simpler and faster classifiers without accuracy losses. Finally, we will define some feedback to facilitate the training phase for the potential users of this kind of system [42]. Different techniques will be analysed in the search for effectiveness to help disabled people obtain control over their brain potentials (waves) and maximise the accuracy of detecting different brain states.

## Supporting information

S1 Dataset Modified BCI competition III—Dataset 3a. Modified dataset including the NC instances for the three subjects: K3b, K6b and L1b.
(ZIP)
